# Calcium Carbonate Addition Improves L-Methionine Biosynthesis by Metabolically Engineered *Escherichia coli* W3110-BL

**DOI:** 10.3389/fbioe.2020.00300

**Published:** 2020-04-24

**Authors:** Hai-Yan Zhou, Wang-Jie Wu, Yue-Ying Xu, Bin Zhou, Kun Niu, Zhi-Qiang Liu, Yu-Guo Zheng

**Affiliations:** ^1^Key Laboratory of Bioorganic Synthesis of Zhejiang Province, College of Biotechnology and Bioengineering, Zhejiang University of Technology, Hangzhou, China; ^2^Engineering Research Center of Bioconversion and Biopurification, Ministry of Education, Zhejiang University of Technology, Hangzhou, China; ^3^The National and Local Joint Engineering Research Center for Biomanufacturing of Chiral Chemicals, Zhejiang University of Technology, Hangzhou, China

**Keywords:** L-methionine, metabolic flux, *Escherichia coli*, calcium carbonate, flux balance analysis

## Abstract

L-Methionine (L-Met) is a sulfur-containing amino acid, which is one of the eight essential amino acids to human body. In this work, the fermentative production of L-Met with genetically engineered *Escherichia coli* W3110-BL in a 5-L fermentor was enhanced through supplement of Ca^2+^ into the fermentation medium. With the addition of 30 g/L calcium carbonate (CaCO_3_), the titer of L-Met and yield against glucose reached 1.48 g/L and 0.09 mol/mol glucose, 57.45% higher than those of the control, respectively. The flux balance analysis (FBA) revealed that addition of CaCO_3_ strengthened the tricarboxylic acid cycle and increased the intracellular ATP concentration by 39.28%. The re-distribution of carbon, ATP, and cofactors flux may collaborate to improve L-Met biosynthesis with *E. coli* W3110-BL. The regulation of citrate synthase and oxidative phosphorylation pathway was proposed to be important for overproduction of L-Met. These foundations provide helpful reference in the following metabolic modification or fermentation control for further improvement of L-Met biosynthesis.

## Introduction

Methionine (Met) is one of the eight essential amino acids of humans. It is extensively applied in the fields of pharmaceutics, food, and feed. Met can be also used as an important participant in the synthesis of *S*-adenosyl methionine (SAM), a common methyl-group donor involved in the biosynthesis of nucleic acids, phospholipids, proteins, epinephrine, melatonin, creatine, and other molecules. Along with L-lysine, Met is a dominant amino acid used in animal feed (Noftsger et al., 2005). In recent years, the global Met market for animal feed has been substantially increased with the rapid growing consumption of meat and milk products.

Currently, microbial fermentation with auxotrophic mutants or genetically engineered strains for production of Met has attracted more attentions than chemical synthesis, which exhibits disadvantages of environmental unfriendliness, strict reaction conditions and impure product with racemic mixtures. The pathway for L-methionine (L-Met) synthesis is well studied in many microorganisms (Krömer et al., 2006). It is a multibranched and multilevel regulated biosynthetic pathway (Huang et al., 2018). L-Met biosynthesis derived from glucose with the canonical metabolic route requires 18 mol ATP, and L-Met is thereby known as the most expensive amino acid in terms of consumed mol of ATP per molecule produced (Kaleta et al., 2013). Large amount of strategies have been developed to improve L-Met fermentative production, as for instance, traditional breeding (Kase and Nakayama, 1975; Kumar et al., 2003), genetical modification (Usuda and Kurahashi, 2005; Park et al., 2007; Huang et al., 2017, 2018), and fermentation optimization (Anakwenze et al., 2014; Zhou et al., 2019). However, the industrial preparation of L-Met by fermentation approach is till restricted since the metabolic flux is dispersed and its regulation is complicated. It is therefore of particular importance to identify the potential factors affecting the L-Met over-production.

In our previous study, based on the comprehensive analysis of feedback inhibition, synthetic bottleneck, and cell growth repression in L-Met biosynthesis, a recombinant L-Met-producing strain *Escherichia coli* IJAHFEBL/pA^∗^H (shortly named as *E. coli* W3110-BL) was constructed via deletion of *metI* for partial inactivation of the L-Met import system MetD, *metJ* for elimination of negative transcription regulation by L-Met, and *lysA* for blocking the biosynthesis of the by-product L-lysine (Huang et al., 2017); additionally, the expression of *metH* encoding B12-dependenthomocysteine-N5-methyltetrahydrofolate transmethylase (MetH), *metF* encoding 5, 10-methylenetetrahydrofolate reductase (MetF), *cysE* encoding serine acetyltransferase (CysE), and *metBL* enconding cystathionine gamma-synthase (MetB)/bifunctional aspartate kinase/homoserine dehydrogenase II (MetL) were enhanced by replacing their native promoters on the chromosome by a strong promoter *trc*, respectively (Huang et al., 2017, 2018). The homoserine *O*-succinyltransferase with reduced feedback sensitivity to SAM and L-Met, as well as the L-Met efflux transporter were overexpressed by constructing a recombinant expression plasmid pA^∗^H, of which the genes *metA*^*fbr*^ and *yjeH* were inserted into the plasmid pTrc99A, respectively (Huang et al., 2018). As a result, the L-Met production was significantly enhanced compared with the original strain *E. coli* W3110.

In order to further enhance the L-Met fermentation titer of this recombinant strain, the fermentation conditions need to be additionally optimized by process engineering. As is well-known, inorganic salts (minerals) are essential elements in the nutrition and are required for almost all living things. Inorganic salts starvation or limitation in microbial cells will lead to critical stress response (Wiesenberger et al., 2007). It has been demonstrated that supplement of optimal mineral salts (for example K_2_HPO_4_, MgSO_4_⋅7H_2_O, NaCl, CaCO_3_, FeSO_4_⋅7H_2_O, MnCl_2_⋅7H_2_O, and ZnSO_4_) in the fermentation medium exhibited positive effects on various fermentation product, such as vitamins (Oraei et al., 2018), amino acids (Zhou et al., 2019), antibacterial metabolite (Jacob et al., 2017), and polypeptide or enzyme (Zhou et al., 2014; Cheng et al., 2019). The bivalent metals including Zn^2+^, Mg^2+^, and Ba^2+^ at trace concentration (mg/L) were reported to play an important role to stimulate L-Met production with *Bacillus thuringiensis* EC1. After optimization of their concentrations, an improved L-Met yield of 3.18 g/L was achieved (Anakwenze et al., 2014). The effect of another bivalent metal calcium ion (Ca^2+^) on cell growth, morphology, and accumulation of target metabolites has also been reported by numerous researchers (Robson et al., 1991; Pera and Callieri, 1997, 1999; Xin and Wang, 2007; Huang et al., 2011). Huang et al. (2011) studied the effect of Ca^2+^ on the fermentation of polyglutamic acid. Addition of calcium chloride resulted in a 15.2% increase in final yield of polyglutamic acid, while the activities of enzymes involved in the metabolism of α-ketoglutaric acid were also improved (Huang et al., 2011). Ca^2+^ in the fermentation broth could also serve as an essential catalytic cofactors to activate the specific enzymes involved in certain metabolic pathways, resulting in re-redirection of the intracellular carbon and energy flux (Liu et al., 2007; Egnatchik et al., 2014).

Flux balance analysis (FBA), as one of the metabolic flux analysis (MFA) approaches, is to analyze the distribution of metabolic flux based on the quantitative equilibrium model of the metabolic network (also be called stoichiometric MFA) (Varma and Palsson, 1994; Papp and Simeonidis, 2007). The input data were mainly derived from the measurement of extracellular flux. The FBA has advantages over the traditional MFA of carbon labeling experiment (CLE) using the isotope labeling such as ^13^C (Ando and Garcia Martin, 2018), owing to the design flexibility and easiness of operation and analysis. As a popular tool for analyzing the metabolic flux, FBA methodology has been rapidly developed and the application has risen over the past more than 30 years, particularly with the purpose of calculation of the maximum theoretical output of metabolism (Krömer et al., 2006; Klamt et al., 2018; Rawls et al., 2019), description of the metabolic flux distribution of the complete central metabolism in industrial strains *Corynebacterium glutamicum* (Vallino and Stephanopoulos, 1993) and *E. coli* (Varma and Palsson, 1994), and estimation of the effects of genetic modification or environmental alteration on intracellular material or energy flux (Li et al., 2017; Zhao et al., 2017; Armingol et al., 2018; Hon et al., 2019; Martinez-Monge et al., 2019).

In this work, calcium was found to be capable of promoting L-Met biosynthesis in the genetically engineered *E. coli* W3110-BL. In order to deeply understand the role of calcium in L-Met metabolic pathway, the quantitative analysis of metabolic flux in the presence of calcium was conducted using FBA. The results were of much importance in developing molecular and engineering strategies for further modifying the L-Met biosynthetic pathway.

## Materials and Methods

### Bacterial Strain and Plasmid

The genetically engineered *E. coli* W3110-BL previously constructed in our laboratory (Huang et al., 2017, 2018) was used as a L-Met producing strain in this study.

### Media and Cultivation Conditions

The seed medium was Luria-Bertani (LB) medium (tryptone 10 g/L, yeast extract 5 g/L, and NaCl 10 g/L) supplemented with 100 μg/mL Amp. The fermentation medium contained 20 g/L glucose, 5 g/L (NH_4_)_2_SO_4_, 1.5 g/L yeast extract, 2 g/L KH_2_PO_4_, 10 g/L Na_2_S_2_O_3_, 1 mg/L Vb_12_, 1 mL/L salt solution (MgSO_4_⋅7H_2_O 0.5 g/L, MnSO_4_⋅8H_2_O 5 mg/L, FeSO_4_⋅7H_2_O 5 mg/L, ZnSO_4_ 5 mg/L), 50 mg/L L-lysine, 100 μg/mL Amp, and 0.1 mmol/L isopropyl β-d-1-thiogalactopyranoside (IPTG). Different amounts of calcium carbonate (CaCO_3_) were supplemented when required.

In batch cultivation for L-Met production, 150 mL of the seed broth after cultivation in LB medium containing 100 μg/L ampicillin for 12 h at 37°C, 180 rpm was inoculated into a 5-L jar fermenter (Winpact Parallel Fermentation System FS-05, Major Science, Taiwan R.O.C) with a working volume of 3 L fermentation medium. The fermentation was initially performed at 37°C with agitation of 300 rpm and aeration of 1.5 vvm. 100 mg/mL Amp was supplemented to maintain plasmid retention as needed. When the optical density of the fermentation broth measured at 600 nm (OD_600_) reached 1.8-2.0, IPTG was added at the final concentration of 0.1 mmol/L and the inducible expression was conducted at 28°C. During the entire fermentation process, the dissolved oxygen (DO) level was maintained above 20% by controlling the agitation rate at 300–500 rpm (Supplementary Figure S1).

### Construction of Metabolic Network of the Engineered *E. coli* W3110-BL

As a workhorse microorganism in biomanufacturing, *E. coli* has been extensively used for the biotechnological production of amino acids. Herein, the strain *E. coli* W3110-BL developed in our previous work by means of genetic manipulations was used for L-Met production.

**FIGURE 1:**
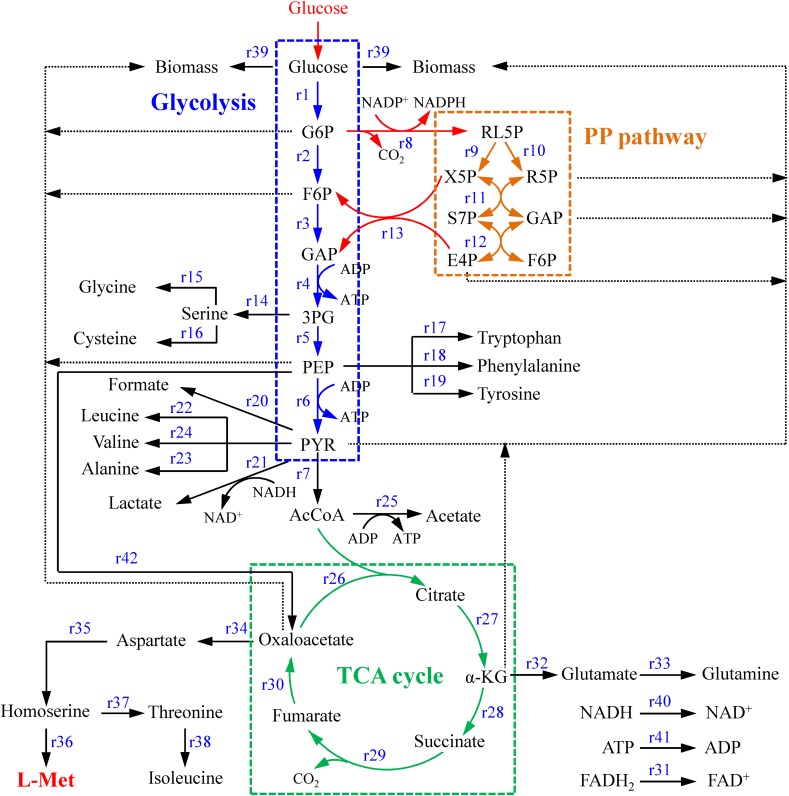
The central metabolism network of *E. coli* W3110-BL. *Abbreviations:* G6P, glucose-6-phosphate; F6P, 6-phosphate fructose; GAP, glyceraldehyde 3-phosphate; 3PG, 3-phosphoglycerate; PEP, phosphoenolpyruvate; PYK, pyruvate; AcCoA, acetyl coenzyme A; α-KG, α-ketoglutarate; RL5P, ribulose-5-phosphate; X5P, xylulose-5-phosphate; R5P, ribose-5-phosphate; E4P, erythrose-4-phosphate; S7P, sedoheptulose-7-phosphate.

The metabolic network of the *E. coli* W3110-BL with glucose as sole carbon source was constructed based on the KEGG metabolic pathway database and the other publications (Koffas et al., 1999; Hong and Lee, 2001), which consisted of 42 reactions and 36 metabolic intermediates (Tables 1, 2; the abbreviation of each metabolite was shown in Supplementary Table S2). As shown in Figure 1, with glucose as carbon source, the carbon flux passed through a series of metabolic pathway or biochemical reactions, mainly including Embden-Meyerhof-Parnas pathway (EMP pathway), pentose phosphate pathway (PP pathway), tricarboxylic acid cycle (TCA cycle), transport reactions, oxidative phosphorylation, biomass formation, and L-Met biosynthesis. For the ease of calculation and analysis, part of the metabolic pathways were simplified according to the reported approaches (He et al., 2014): (1) the amino acid degradation reaction, the nucleotide salvage pathway, and other carbon source utilization pathways were omitted; (2) The routes of other amino acids metabolism were not included, except for the essential biosynthesis pathway of the amino acids detectable in the fermentation broth; (3) The cell composition (biomass) was simplified to consist of proteins, DNA, RNA, fatty acids, and cell walls; and the synthesis of fatty acids and cell walls was expressed as the precursors synthesis in the metabolic network; (4) Since the branch pathway for L-lysine formation had been knocked out during the construction process of the engineered strain (Huang et al., 2017), the synthetic route of L-lysine was not considered in this study.

**TABLE 1:** The metabolic reaction of the engineered *E. coli* W3110-BL.

**No.**	**Metabolic reaction**
**Glycolysis**	
1	GLC + ATP → G6P + ADP
2	G6P ↔ F6P
3	F6P + ATP → GAP + ADP
4	GAP + ADP + NAD^+^ ↔ 3PG + ATP + NADH
5	3PG ↔ PEP
6	PEP + ADP → PYR + ATP

**Pentose phosphate pathway**	
8	G6P + 2 NADP^+^→ RL5P + 2 NADPH + CO_2_
9	RL5P ↔ X5P
10	RL5P ↔ R5P
11	X5P + R5P ↔ GAP + S7P
12	S7P + GAP ↔ F6P + E4P
13	X5P + E4P → F6P + GAP

**TCA cycle**	
7	PYR + NAD^+^→AcCoA + CO_2_ + NADH
26	OXA + AcCoA ↔ CIT
27	CIT + NADP^+^ ↔α-KG + CO_2_ + NADPH
28	α-KG + ADP + NAD^+^ ↔ SUC + CO_2_ + ATP + NADH
29	SUC + FAD^2+^ ↔ FUM + FADH_2_
30	FUM + NAD^+^ ↔ OXA + NADH
42	PEP + CO_2_→ OXA

**Amino acid biosynthesis**	
14	3PG + Glu + NAD^+^→ Ser + α-KG + NADH
15	Ser + 2 NAD^+^→Gly + CO_2_ + 2 NADH
16	Ser + SO_4_^2–^ + 4 ATP + 4 NADPH →Cys + 4 ADP + 4 NADP^+^
17	E4P + 2 PEP + R5P + Gln + Ser + 3 ATP + NADPH →Trp + PYR + Glu + GAP + CO_2_ + NADP^+^ + 3 ADP
18	E4P + 2 PEP + Glu+ ATP + NADPH →Phe + Gln + α-KG + CO_2_ + ADP + NADP^+^
19	E4P + 2 PEP + Glu + ATP + NADPH + NAD^+^→ Tyr + Gln + α-KG + CO_2_+ ADP + NADP^+^ + NADH
22	2 PYR + AcCoA + Gln + NADPH + NAD^+^→ Leu + α-KG + 2 CO_2_+ NADH + NADP^+^
23	PYR + NH_4_^+^ + NADH → Ala + NAD^+^
24	2 PYR + Glu + NADPH ↔ Val + α-KG + CO_2_ + NADP^+^
32	α-KG + NH_4_^+^ + NADPH ? Glu + NADP^+^
33	Glu + NH_4_^+^ + ATP →Gln + ADP
34	OXA + Glu → Asp + α-KG
35	Asp + ATP + NADPH →Hom + NAD^+^
36	Hom + Cys→ Met + PYR+ NH_4_^+^ + SUC
37	Hom + ATP →Thr + ADP
38	Thr + Glu + PYR + NADPH → Ile + α-KG + CO_2_ + NH_4_^+^ + NADP^+^

**Transport reactions**	
20	PYR + CoA →AcCoA + FOR
21	PYR + NADH ↔ NAD^+^ + LAC
25	AcCoA + ADP → ACE + ATP

**Oxidative phosphorylation/maintenance energy**	
31	FADH_2_ + 1/2 O_2_ + 2 ADP → 2 ATP + FAD^2+^
40	NADH + 1/2 O_2_ + 3 ADP → 3 ATP + NAD^+^
41	ATP → ADP + Pi

**Biomass formation**	
39	0.488 Ala + 0.229 Asp + 0.087 Cys + 0.250 Glu + 0.250 Gln + 0.582 Gly + 0.428 Leu + 0.146 Met + 0.176 Phe + 0.241 Thr + 0.054 Trp + 0.131 Tyr + 0.402 Val + 0.205 G6P + 0.071 F6P + 0.754 R5P + 0.129 GAP + 0.619 3PG + 0.276 Ile + 0.051 PEP + 0.083 PYR + 2.510 AcCoA + 0.087 α-KG + 0.340 OXA + 33.247 ATP + 5.363 NADPH → Biomass + 1.455 NADH
Biomass: C_1_H_1.8_O_0.5_N_0.2_	

**TABLE 2:** The metabolites of *E. coli* W3110-BL and the reaction equations.

**No.**	**Metabolites**	**Equation**
1	ATP	− r1 − r3 + r4 + r6 − 4 r16 − 3 r17 − r18 − r19 + r25 + r28 + 2 r31 − r33 − r35 − r37 − 33.247 r39 + 3 r40 − r41
2	NADH	r4 + r7 + r14 + 2 r15 + r19 − r21 + r22 − r23 + r28 + r30 + 1.455 r39 − r40
3	NADPH	2 r8 − 4 r16 − r17 − r18 − r19 − r22 − r24 + r27 − r32 − r35 − r38 − 5.363 r39
4	FADH_2_	r29 − r31
5	G6P	r1 − r2 − r8 − 0.205 r39
6	F6P	r2 − r3 + r12 + r13 − 0.071 r39
7	3PG	r4 − r5 − r14 − 0.619 r39
8	PEP	r5 − r6 − 2 r17 − 2 r18 − 2 r19 − 0.051 r39 − r42
9	PYR	r6 − r7 + r17 − r20 − r21 − 2 r22 − r23 − 2 r24 − r38 − 0.803 r39
10	AcCoA	r7 + r20 − r22 − r25 − r26 − 2.510 r39
11	CIT	r26 − r27
12	RL5P	r8 − r9 − r10
13	X5P	r9 − r11 − r13
14	R5P	r10 − r11 − r17 − 0.754 r39
15	S7P	r11 − r12
16	GAP	2 r3 − r4 + r11 − r12 + r13 + r17 − 0.129 r39
17	E4P	r12 − r13 − r17 − r18 − r19
18	α-KG	r14 + r18 + r19 + r22 + r24 + r27 − r28 − r32 + r34 + r38 − 0.087 r39
19	SUC	r28 − r29 + r36
20	FUM	r29 − r30
21	OXA	− r26 + r30 − r34 − 0.34 r39 + r42
22	Asp	r34 − r35 − 0.229 r39
23	Hom	r35 − r36 − r37
24	Thr	r37 − r38 − 0.241 r39
25	Glu	− r14 + r17 − r18 − r19 − r24 + r32 − r33 − r34 − r38 − 0.25 r39
26	Ala	r23 − 0.488 r39
27	Cys	r16 − 0.087 r39
28	Gln	− r17 + r18 + r19 − r22 + r33 − 0.25 r39
29	Gly	r15 − 0.582 r39
30	Leu	r22 − 0.428 r39
31	Met	r36 − 0.146 r39
32	Phe	r18 − 0.176 r39
33	Tyr	r19 − 0.131 r39
34	Val	r24 − 0.402 r39
35	Ser	r14 − r15 − r16 − r17
36	Ile	r38 − 0.276 r39

### Theory of FBA

Metabolic networks are non-linear complex networks regulated by kinetic mechanisms, usually involving hundreds of metabolites and thousands of reactions. Constructing genome-scale metabolic models manually is time-consuming and labor-intensive. Therefore, it is necessary to build mathematical models of these metabolic networks to describe, understand, and eventually predict the system metabolic behavior (Tran et al., 2008). Generally, the metabolic network is constructed by aggregating the reported data in various literature and databases. After construction of the metabolic network, the flux distribution of the metabolic network is to be determined.

In order to elucidate the reasons for the improvement of L-Met production by addition of CaCO_3_, a metrological model FBA, based on mass conservation and pseudo-steady-state (PSS) of metabolic intermediates (Stephanopoulos and Sinskey, 1993), was adopted to estimate the effects of CaCO_3_ on metabolic flux distribution of the *E. coli* W3110-BL. First, assuming that the net change in the concentration of either reactants or products in the metabolic network was 0, and the reaction was in a state of equilibrium. There were *n* constraints on reaction rates change derived from *n* intermediates. Then assuming the total number of rates needed to be determined is *J*, so the degree of freedom was *F* = *J* – *n.*

The flux distribution of the complete metabolic network can be determined by measuring the extracellular metabolites only if their number is more than the degree *F*. For each metabolite, the mass balance can be expressed as:

(1) $$\frac{d\,X_{i}}{d\,t}=\sum_{j}S_{i\,j}\,V_{j}$$

where, *S*_*ij*_ in the metrology matrix represents the number of moles of metabolite *i* that produced or consumed in reaction *j*. *V*_*j*_ is the *j*th flux in the system; *X*_*i*_ stood for the concentration of metabolite.

When the metabolic network is at steady state, according to the principle of mass balance, the total inputting flux of each metabolite in the network toward its metabolic pool is equal to the output, so the formula could be reduced to a set of homogeneous linear equation, described in matrix notation as:

(2) S⋅v=0⁢

Where, *S* was an *m* × *n* metrology matrix. *m* was the number of metabolites and *n* the number of reactions in the system. *v* was an *m*-dimensional metabolic reaction rate vector.

In this study, the constructed model included 42 metabolic reactions and 36 metabolites (Figure 1). The freedom degree of the matrix was six. The specific cell growth rate (r39), specific glucose consumption rate (r1), as well as the specific accumulation rates of formic acid (r20), lactic acid (r21), acetic acid (r25), succinic acid (r28), and L-Met (r36), were determined based on the extracellular measurement. The positive definite matrix was calculated by Matlab 2016a software (Morales et al., 2016). All fluxes were converted to percentages based on a glucose uptake rate of 100 mmol/g cell/h.

### Analytical Methods

The extracellular concentrations of pyruvic acid, formic acid, lactic acid, acetic acid, α-ketoglutarate, and succinic acid were determined by high-performance liquid chromatography (HPLC) (Waters, Milford, MA, United States) equipped with a RID detector (Waters 2410, Waters, Milford, MA, United States). For metabolites separation, an Aminex HPX-87H column (BioRad, München, Germany) was used, working at 35°C and a flow rate of 0.6 mL/min with 5 mmol/L sulfuric acid as mobile phase. Biomass, residual glucose, and L-Met was measured as described previously (Zhou et al., 2019). The specific oxygen uptake rate was measured by the dynamic method (Santos et al., 2006). The specific carbon dioxide production rate was determined by a titrimetric method (Converti et al., 2003).

For preparation of cell-free extracts, the *E. coli* cell pellets were collected, washed and suspended in 100 mM potassium phosphate buffer (pH 7.0). The pellets were disrupted by sonication (work time 5 s, interval 10 s, total time 2 min) in an ice bath using an ultrasonic processor VCX 130PB (Sonic & Materials Inc., Newtown, United States). Cellular debris was removed by centrifugation at 12,000 *g* and 4°C for 5 min. The crude extracts were used for assays of enzyme activity and intracellular calcium irons level. Protein concentration was determined by the Bradford method (Bradford, 1976) using bovine serum albumin as the standard.

A Citrate Synthase Activity Assay Kit (Nanjing Jiancheng Bioengineering Institute, Nanjing, China) was used for Citrate synthase (CS) activity detection. According to the instruction of Kit, CS activity was assayed *spectrophotometrically* at 412 nm using 5,5′-dithiobis (2-nitrobenzoic acid) (DTNB) colorimetric assay method (Srere, 1969; Quandt et al., 2015).

The calcium concentration was assayed using a Calcium Assay Kit (Nanjing Jiancheng Bioengineering Institute, Nanjing, China) with methylthymol blue (MTB) as a color agent (Themelis et al., 1999). The absorbance of the colored Ca-MTB complex produced was monitored at 610 nm by a Microplate Spectrophotometer SpectraMax M2 (Molecular Devices, CA, United States).

The transcriptional responses of the genes associated with the TCA cycle to CaCO_3_ addition were detected by the qRT-PCR. Samples were taken at the logarithmic growth phase (15 h) during the fermentation process with or without addition of CaCO_3_ and treated by flash-freezing in liquid nitrogen. The total RNA was extracted using RNAiso Plus reagent (Takara, Kyoto, Japan) according to the manufacturer’s instructions. The RNA quality was determined using 1% agarose gel electrophoresis and the quantity was measured with a NanoDrop 2000c UV–vis Spectrophotometer (Thermo Scientific, Madison, United States). Each RNA sample was reverse-transcribed to cDNA by the PrimeScript^TM^ RT reagent Kit (Takara, Kyoto, Japan) according to the manufacturer’s manual. The qRT-PCR was carried out on a LightCycler 480 System (Roche, Basel, Switzerland) using TB Green real-time PCR mix (Takara, Kyoto, Japan) with the specific primers (Supplementary Table S1). The house-keeping gene 16S rRNA was used as an internal control. The PCR amplification was performed in 10 μL reaction mixtures, which consisted of 5 μL 2 × SYBR Premix Ex Taq, 0.5 μL diluted cDNA, 0.2 μL forward and reverse primer, and 4.1 μL RNase free water. All the PCR reactions were carried out using the following protocol: 95°C for 30 s; 40 cycles of 94°C for 3 s and 60°C for 30 s. The qRT-PCR for each sample was performed in triplicate and the results were analyzed with the 2^–ΔΔ*Ct*^ method (Livak and Schmittgen, 2001).

### Statistical Analysis

All experiments were carried out in triplicate and the average values were reported. The values of average and standard deviation (SD) were calculated in Microsoft Excel. The statistical differences were determined according to student’s *t*-test. *P* values of <0.05 were considered statistically significant.

## Results and Discussion

### CaCO_3_ Addition Improves L-Met Biosynthesis in the Engineered *E. coli* W3110-BL

**FIGURE 2:**
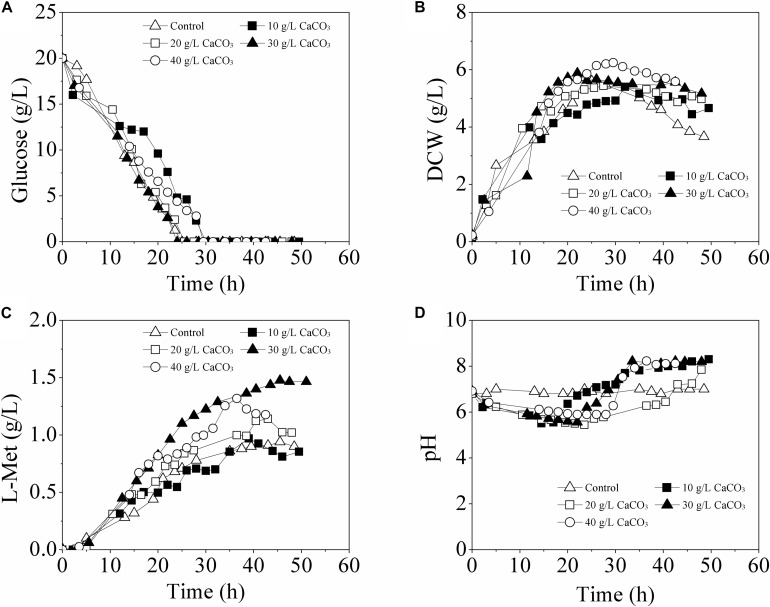
Time course of fermentative production of L-Met with *E. coli* W3110-BL in 5-L fermentor supplemented with different amounts of CaCO_3_. **(A)** glucose consumption; **(B)** cell growth; **(C)** L-Met biosynthesis; **(D)** pH variation. All measurements were performed in triplicate. For clarity, the error bars were not displayed.

For L-Met biosynthesis, the engineered *E. coli* W3110-BL was grown in batch cultures performed in 5-L fermentor, during which the pH of the fermentation medium was maintained at 6.8 ± 0.2 either with 30% NH_3_⋅H_2_O or with CaCO_3_ in different concentrations (10, 20, 30, and 40 g/L). As shown in Figure 2, when the pH was adjusted with 30% NH_3_⋅H_2_O, only 0.94 g/L L-Met was accumulated; while with CaCO_3_ as pH regulator, L-Met accumulation was enhanced compared with that of the control (with 30% NH_3_⋅H_2_O). Furthermore, L-Met titer was increased as the CaCO_3_ amount raised from 0 to 30 g/L, with the maximum value of 1.48 g/L at 30 g/L CaCO_3_ (57.45% higher than that of the control). The yield of L-Met against glucose was 0.089 mol/mol, considerably increased compared with that of the control (0.057 mol/mol). However, with CaCO_3_ further increased to 40 g/L, decreased L-Met production was observed. Therefore, from the view of fermentation optimization, 30 g/L CaCO_3_ was determined to be most conducive to L-Met biosynthesis.

It should be noted that, when using CaCO_3_ as pH regulator, the pH of the fermentation medium was not constant. As was seen from Figure 2D, in batch cultivation with different amounts of CaCO_3_ addition, the pH was approximately at 6.8 (set value) in the initial stage and slowly varied (first decreased and then increased) between 5.5 and 8.2 with different patterns till the end of the fermentation.

To clarify whether pH or the presence of calcium really affected the L-Met production, other two different modes of batch fermentation were also tried. In Mode I, the pH was maintained at 6.8 ± 0.2 by automatic addition of 30% NH_3_⋅H_2_O as well as supplement of 30 g/L CaCO_3_; in Mode II, CaCl_2_ (33.3 g/L) was used to displace CaCO_3_. Herein, the mode with pH controlled at 6.8 ± 0.2 by automatic addition of 30% NH_3_⋅H_2_O was named as Control-I, and the one only with 30 g/L CaCO_3_ as pH regulator was named as Control-II. Except for the pH in Control-II that was varied between 5.5 and 8.2 over time (Figure 2D), the pH values in Control-I, Mode I, and Mode II were all well controlled at about 6.8 (data not shown).

**FIGURE 3:**
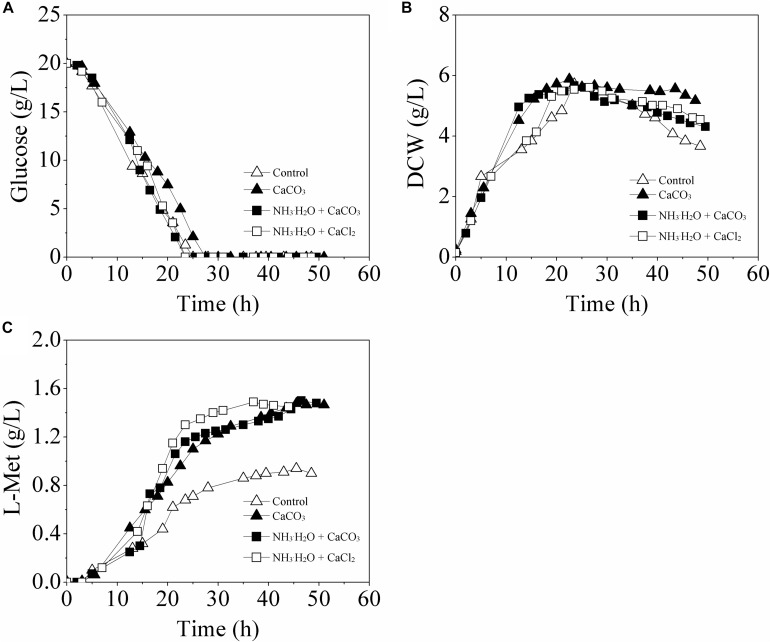
Time course of L-Met fermentation by the genetically engineered *E. coli* W3110-BL in batch cultures with different pH control modes. **(A)** glucose consumption; **(B)** cell growth; **(C)** L-Met biosynthesis. All measurements were performed in triplicate. For clarity, the error bars were not displayed.

As shown in Figure 3, with different modes of pH control, the profiles of glucose consumption and cell growth exhibited no crucial distinction among all the batch runs. Interestingly, when the pH was maintained at 6.8 ± 0.2 by 30% NH_3_⋅H_2_O, either with addition of CaCO_3_ (Mode I) or CaCl_2_ (Mode II), the L-Met accumulation was also greatly improved, 59.57 and 58.51% higher than that of the Control-I, respectively.

The fermentation patterns in Control-II and Mode I which both contained 30 g/L CaCO_3_ were compared and analyzed further. Interestingly, although the extracellular pH values of *E. coli* cells in these two cases were different, there was no significant difference with respect to L-Met biosynthesis. This indicated that calcium ions rather than environmental pH were most responsible for the enhancement of L-Met accumulation.

**FIGURE 4:**
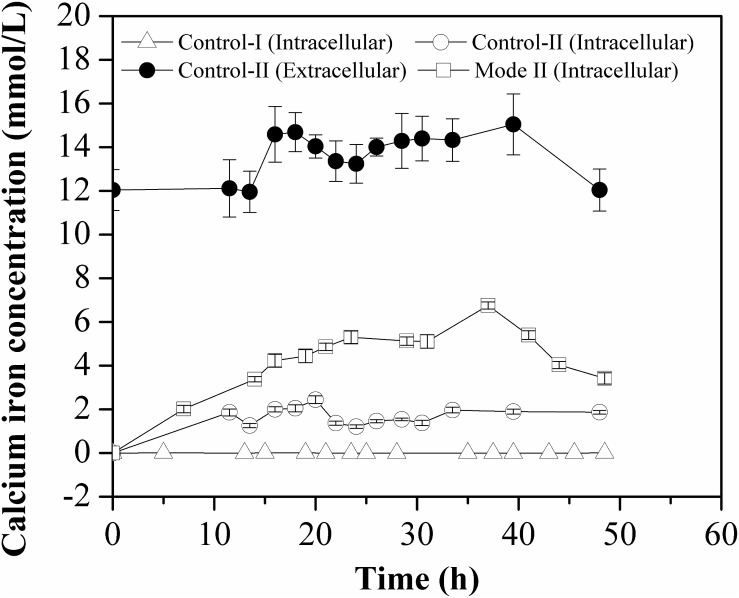
Free Ca^2+^ concentration inside the cells grown in different batch cultures. The intracellular Ca^2+^ concentration was measured in crude cell extracts and all measurements were performed in triplicate.

As shown in Figure 4, the intracellular calcium concentrations in Control-II and Mode II were in the same order of magnitude, although the solubility of CaCO_3_ is much lower than that of CaCl_2_ in aqueous solution. And it was considerably higher in each mode (1–10 mmol/L) in comparison with that in Control-I (0.5–8.0 μmol/L), implying that the consumption of calcium ions (intracellular free Ca^2+^ concentration) was highly correlated with the accumulation of L-Met.

### By-Products Formation in the Batch Fermentation With CaCO_3_ Addition

**FIGURE 5:**
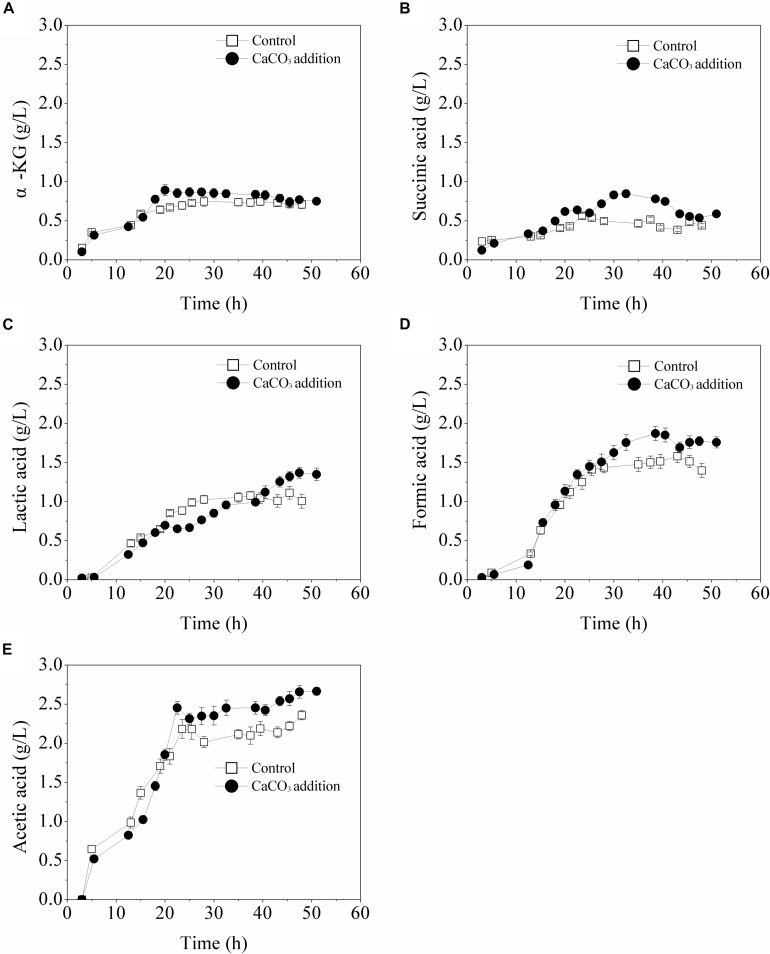
Biosynthesis of organic acids in batch cultures in 5-L fermentor without or with CaCO_3_ (30 g/L) addition. SD is shown for triplicate measurements of each organic acid. **(A)** α-KG, **(B)** succinic acid, **(C)** lactic acid, **(D)** formic acid, and **(E)** acetic acid.

The formed by-products were detected and the results were illustrated in Figure 5. With the existence of CaCO_3_, the maximum concentrations of main organic acids including α-ketoglutarate, succinic acid, lactic acid, formic acid, and acetic acid were increased to different degrees. As for α-ketoglutarate and succinic acid, the highest accumulation in the case of CaCO_3_ addition was increased by 50 and 20.3%, respectively, compared with that of the control. It was speculated that the presence of CaCO_3_ probably increased the carbon flux of the TCA cycle. After the glucose was exhausted, the accumulations of α-ketoglutarate and succinic acid were both decreased, likely due to their conversion to other metabolic products. By contrast, the biosynthesis of formic acid, lactic acid, and acetic acid was not immediately influenced by glucose limitation in the presence of CaCO_3_. All the main organic acids formed in the fermentation process might be re-utilized for maintenance of cell growth or L-Met biosynthesis in the late fermentation phase.

### The Effect of CaCO_3_ Addition on Carbon Metabolic Flux Distribution

In order to reveal the specific role of CaCO_3_ addition on the L-Met biosynthesis pathway in *E. coli* W3110-BL, the intracellular metabolic flux distribution was estimated using the FBA method. 12 reaction rates including the specific consumption rate of glucose (r1)/oxygen and the specific formation rate of formic acid (r20), lactic acid (r21), acetic acid (r25), α-ketoglutarate, succinic acid (r28), L-Met (r36), biomass (r39), carbon dioxide, pyruvic acid, and other amino acids (L-isoleucine, L-leucine, L-threonine, L-homoserine, and L-tyrosine) were calculated and summarized in Table 3. The other amino acids were expressed as a homogenized chemical with a formula of C_5_H_11_NO_2_._6_.

**TABLE 3:** Rate parameters of metabolic reaction in *E. coli* W3110-BL.

**Fermentation parameters**	**Chemical formula**	**Control**	**CaCO_3_ addition**
**Specific consumption rate (mmol/g DCW/h)**			
Glucose (r1)	C_6_H_12_O_6_	0.95 ± 0.056	0.77 ± 0.022
Oxygen	O_2_	1.330 ± 0.098	1.08 ± 0.045
**Specifc formation rate (mmol/g DCW/h)**			
Formic acid (r20)	CH_2_O_2_	0.41 ± 0.017	0.33 ± 0.020
Lactic acid (r21)	C_3_H_6_O_3_	0.10 ± 0.007	0.04 ± 0.003
Acetic acid (r25)	C_2_H_4_O_2_	0.32 ± 0.010	0.37 ± 0.018
α-Ketoglutarate	C_5_H_6_O_5_	0.02 ± 0.001	0.09 ± 0.004
Succinic acid (r28)	C_4_H_6_O_4_	0.14 ± 0.010	0.19 ± 0.002
L-Met (r36)	C_5_H_11_O_2_NS	0.06 ± 0.003	0.07 ± 0.004
Cell growth (h^–1^) (r39)	C_1_H_1_._8_O_0_._5_N_0_._2_	0.03 ± 0.002	0.02 ± 0.004
Carbon dioxide	CO_2_	1.25 ± 0.063	0.98 ± 0.033
Pyruvic acid	C_3_H_4_O_3_	0.11 ± 0.010	0.02 ± 0.003
Other amino acids^*a*^	C_5_H_11_NO_2_._6_	0.08 ± 0.002	0.02 ± 0.001
**Carbon balance (%)**		97.4 ± 1.83	102.1 ± 2.44
*Reduction degree (%)*		94.4 ± 3.60	99.0 ± 3.29

*^*a*^Other amino acids included L -isoleucine, L-leucine, L-threonine, L-homoserine, and L-tyrosine. For convenience, these amino acids were expressed as a homogenized chemical with a formula of C_5_H_11_NO_2_._6_.*

Carbon recoveries as biomass and various metabolites were 97.4–102.1% of the theoretical maximum, suggesting that the major metabolites were properly identified and quantified. The reduction degree calculated based on the equation ε = 4 C + H − 2 O − 3 N (Oh et al., 2008) were well balanced. Among these reaction rates, r1, r20, r21, r25, r28, r36, and r39 were used to determine the volumetric formation rates of intracellular metabolites.

**FIGURE 6:**
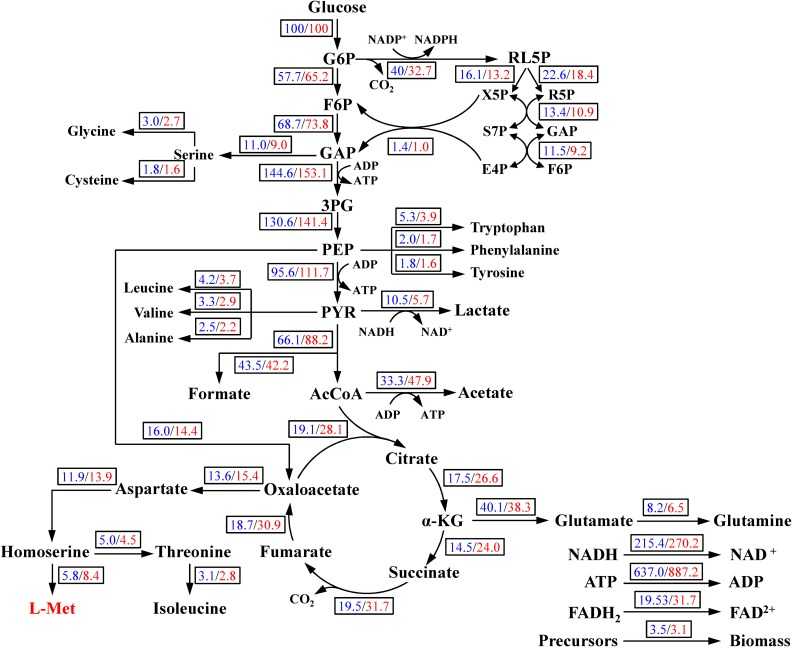
Metabolic flux distributions in the central metabolic pathways at 19 h in the case of control/CaCO_3_ addition. Fluxes shown are normalized to glucose uptake rate of 100 for each condition. All data were in mmol/g DCW/h except biomass g/g DCW/h.

The metabolic model included the main biochemical pathways in carbon central metabolism: glycolysis, PP pathway, TCA cycle, amino acid synthesis, transport reactions, oxidative phosphorylation, and biomass formation. The average intracellular metabolic flux distribution in the late logarithmic growth phase (19 h) was calculated and shown in Figure 6. This model developed herein was simple but biologically meaningful in description of the central metabolism and L-Met biosynthesis, thus the quality of the outcome computed from the model was valid in estimating the effect of CaCO_3_ addition on *E. coli* intracellular flux distributions in batch cultures.

As shown in Figure 6, carbon flux in the central metabolism was redistributed in response to CaCO_3_ addition. The carbon flux into L-Met biosynthesis was increased by 44.83% in the case of CaCO_3_ addition, whereas the carbon flux into the TCA cycle was also increased considerably. Under the aerobic condition, the glycolysis and TCA cycle were correlated by the conversion of PEP to oxaloacetate catalyzed by phosphoenolpyruvate carboxylase as well as the formation of citrate from oxaloacetate and acetyl-CoA catalyzed by the citrate synthase. It could be seen from Figure 6 that the carbon flux into oxaloacetate from PEP was slightly varied, while the flux for citrate biosynthesis from oxaloacetate and acetyl-CoA was increased by 46.67%. Therefore, the increase of carbon flux in the TCA pathway was probably achieved by directing the metabolites flux into citrate biosynthesis from the pool of PEP, pyruvate, and the acetyl-CoA.

**FIGURE 7:**
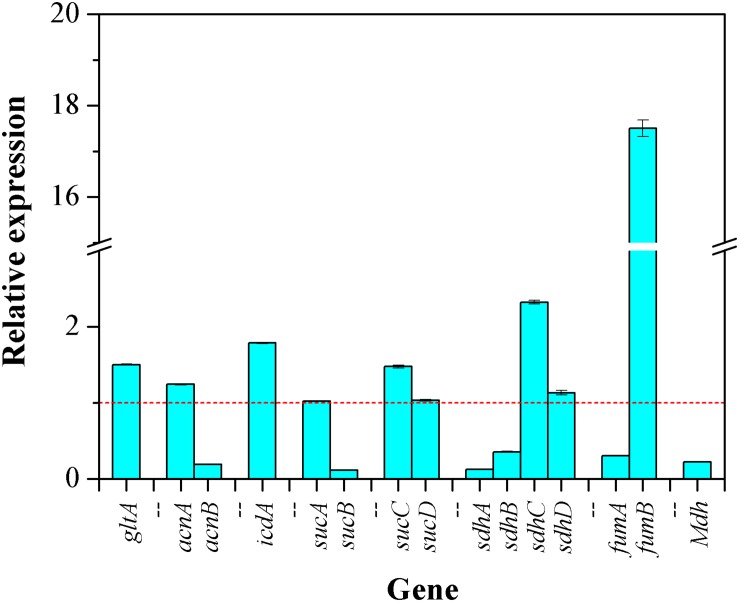
The transcriptional responses of the genes involved in TCA cycle to CaCO_3_ addition. The dashed line represented the relative fold change of gene expression of in the control (the value is 1.0).

With CaCO_3_ addition, the relative changes in transcription levels of 15 genes involved in TCA cycle were determined by qRT-PCR. As shown in Figure 7, the transcriptional level of 7 genes including *gltA* encoding CS, *acnA* encoding aconitase (ACO) isoenzyme, *icdA* encoding isocitrate dehydrogenase (ICDH), *sucC* encoding succinyl-CoA synthetase (SCS) isoenzyme, *sdhC*/*sdhD* encoding succinate dehydrogenase (SDH) isoenzymes, and *fumB* encoding fumarase (FH) isoenzyme were higher than those of the control (without CaCO_3_ addition), indicating an up-regulated expression of these genes. By contrast, the expressions of 6 genes including *acnB* encoding ACO isoenzyme, *sucB* encoding α-ketoglutarate dehydrogenase (KGDH) isoenzyme, *sdhA*/*sdhB* encoding SDH isoenzymes, *fumA* encoding FH isoenzyme, and *mdh* encoding malate dehydrogenase (MDH) were down-regulated. While, there were no significant changes in the relative transcriptional quantitation of *sucA* encoding KGDH isoenzyme and *sucD* encoding SCS isoenzyme. Although the transcriptional responses of these 15 genes to CaCO_3_ supplement were different, it could be certain that, the expression of CS, ICDH, and SCS were enhanced due to the presence of CaCO_3_.

**FIGURE 8:**
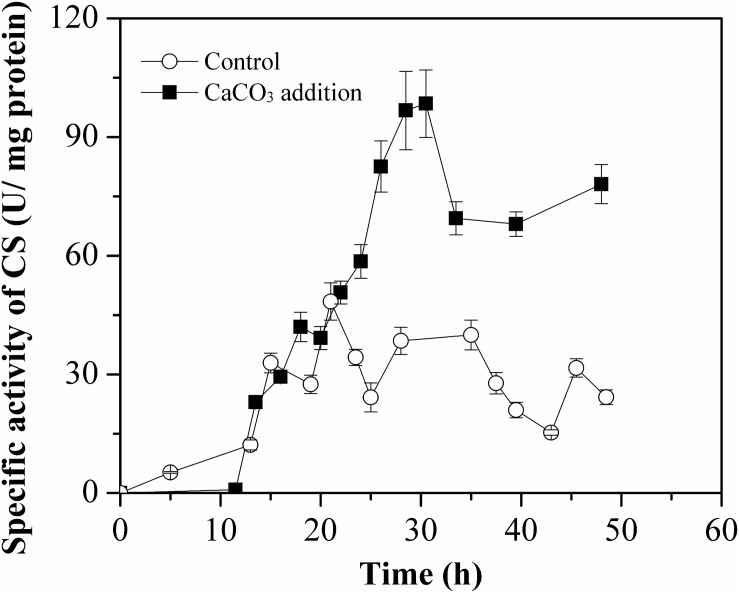
Specific activities of citrate synthase (CS) of *E. coli* W3110-BL during growth in the batch cultivation with or without CaCO_3_ addition. The CS activity was measured in crude cell extracts and all measurements were performed in triplicate.

To evaluate the effect of Ca^2+^ on intracellular CS activity during L-Met biosynthesis, CS specific activity was monitored in cell-free crude extract by means of DTNB colorimetric assay method. As illustrated in Figure 8, the activation of CS by Ca^2+^ before 15 h was not observed. However, the specific activity of CS was much higher than that of the control after 15 h, especially during 22–48 h. The increase in CS activity was allowed for perturbation of the acetyl-CoA branch point with enhanced TCA influx in the presence of CaCO_3_.

The results indicated that, CaCO_3_ addition could direct more carbon to participate in L-Met biosynthesis, through fluctuation of the carbon balance in some essential metabolic pathways, particularly in the TCA cycle. CS, responsible for the entry reaction of a two-carbon unit into the TCA cycle, was thus considered to be a promising metabolic control node for L-Met overproduction.

### The Effect of CaCO_3_ Addition on ATP and Cofactor Metabolism

**FIGURE 9:**
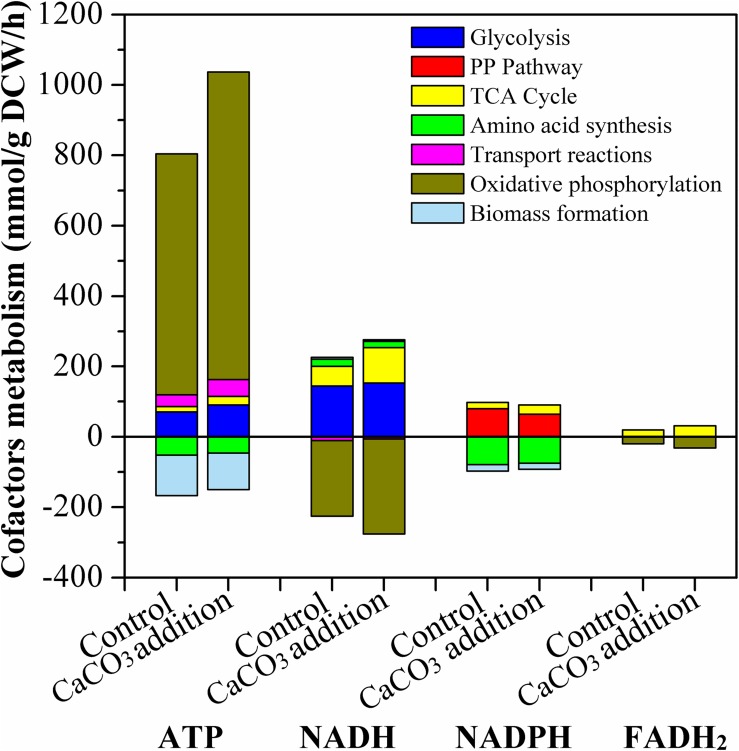
Estimated production and consumption of ATP, NADH, NADPH, and FADH_2_ by the *E. coli* W3110-BL without or with CaCO_3_ addition. All data represent the average value of three estimation. For clarity, the error bars were not shown. The hydrolysis of ATP to ADP was not included in this figure.

The formation and consumption of ATP, NADH, NADPH, and FADH_2_ in the related biochemical pathways was estimated and presented in Figure 9. ATP was mainly produced through glycolysis, TCA cycle, transport reactions pathways, and oxidative phosphorylation; and it was consumed by amino acid synthesis and biomass formation. The ATP formation in the presence of CaCO_3_ was obviously improved compared with the condition of CaCO_3_ absence. ATP was generated at a relative high level, particularly in the oxidative phosphorylation pathway. Therefore, CaCO_3_ addition could provide more energy for L-Met biosynthesis.

The major pathway of NADPH supply included PP pathway and TCA cycle, and NADPH was mainly consumed by amino acid synthesis and biomass formation. Different from NADPH, NADH was synthesized through glycolysis and TCA cycle, and oxidative phosphorylation was its dominant consumption pathway. The main source of FADH_2_ was TCA cycle and it was solely used for oxidative phosphorylation. These results were in good agreement with those of other studies (Kind et al., 2013; He et al., 2014). As Figure 9 indicated, Ca^2+^ could regulate the intracellular cofactor levels, through increasing the generation of NADPH, NADH and FADH_2_ in TCA cycle, or increasing the consumption of NADH and FADH_2_ in oxidative phosphorylation.

NADH, FADH_2_, and NADPH are the key cofactors (or coenzymes) in the cellular metabolic network. NADH and FADH_2_ function as electron carriers and participate in almost all oxidation-reduction reactions. They play crucial roles in cellular energy generation in the living cells. Generally, the ATP yielded during direct glucose metabolism is very limited, while both NADH and FADH_2_ involved in the process of cellular respiration can help in producing more ATP through electron transfer and oxidative phosphorylation reaction. NADPH usually acts as a hydrogen donor to participate in various metabolic reactions (Kind et al., 2013); Besides, it affects numerous enzymatic activities and endogenous concentrations of regulators in many chemical reactions (Xu et al., 2018), such as the synthesis pathway of lipids, fatty acids, and nucleotides. Therefore, the level of cofactors NADH, FADH_2_, and NADPH are essential to amino acid biosynthesis via microbial fermentation (Singh et al., 2007). It has been proved that, increasing the supply of NADPH has a positive effect on the fermentative production of amino acids (Sharp, 2013; Wang et al., 2013). Furthermore, based on regulation of the intracellular cofactor level, optimization of cellular physiological and metabolic functions to maximize the carbon flux distribution and energy supply for the biosynthetic pathway of several kinds of amino acids has been achieved (Bartek et al., 2010; Yin et al., 2014; Xu et al., 2018).

As a summary, exogenous addition of CaCO_3_ could regulate the oxidative phosphorylation pathway to produce more ATP. Additionally, the intracellular cofactors concentration also varied due to CaCO_3_ existence. These results suggested that the improvement of L-Met biosynthesis was correlated with ATP and cofactor metabolism, and was likely due to the collaborative contribution of these effects. It provided an important reference in metabolic engineering to channel more energy flux into the synthesis of target amino acid.

## Conclusion

The positive impact of Ca^2+^ on L-Met biosynthesis of the engineered *E. coli* W3110-BL was demonstrated through comparative assay of different modes of batch fermentation with or without Ca^2+^ addition into the fermentation medium. Intracellular metabolic flux distribution and the metabolism of ATP and cofactors in response to Ca^2+^ existence at the late logarithmic growth phase (19 h) were estimated by FBA. The results indicated that addition of CaCO_3_ (30 g/L) into the fermentation medium resulted in an increased metabolic flux in TCA cycle and a relatively high level of ATP and cofactors generation, which may collaboratively contribute to the enhancement of L-Met biosynthesis. This study lay important fundament for further modification of the potential metabolic control node to channel more metabolic flux into L-Met over-production.

## Data Availability Statement

The raw data supporting the conclusions of this article will be made available by the authors, without undue reservation, to any qualified researcher.

## Author Contributions

H-YZ, W-JW, KN, Z-QL, and Y-GZ participated in designing of work. The analysis and interpretation of experimental the data was jointly conducted by all authors. H-YZ and W-JW prepared the manuscript with the help of Z-QL. W-JW, Y-YX, and BZ performed the experiments.

## Conflict of Interest

The authors declare that the research was conducted in the absence of any commercial or financial relationships that could be construed as a potential conflict of interest.

## References

[B1] Anakwenze V. N. Ezemba C. C. Ekwealor I. A. (2014). Optimization of fermentation conditions of *Bacillus thuringiensis* EC1 for enhanced methionine production. *Adv. Microbiol.* 4 344–352. 10.4236/aim.2014.47041

[B2] Ando D. Garcia Martin H. (2018). “Two-scale 13C metabolic flux analysis for metabolic engineering,” in *Synthetic Metabolic Pathways*, 1st Edn, eds Jensen M. K. Keasling J. D. (New York, NY: Humana Press), 333–352. 10.1007/978-1-4939-7295-1_21 29170969

[B3] Armingol E. Tobar E. Cabrera R. (2018). Understanding the impact of the cofactor swapping of isocitrate dehydrogenase over the growth phenotype of *Escherichia coli* on acetate by using constraint-based modeling. *PLoS One* 13:e0196182 10.1371/journal.pone.0196182 PMC5909895 29677222

[B4] Bartek T. Blombach B. Zonnchen E. Makus P. Lang S. Eikmanns B. J. (2010). Importance of NADPH supply for improved L-valine formation in *Corynebacterium glutamicum*. *Biotechnol. Prog.* 26 361–371. 10.1002/btpr.345 20014412

[B5] Bradford M. M. (1976). A rapid and sensitive method for the quantitation of microgram quantities of protein utilizing the principle of protein-dye binding. *Analyt. Biochem.* 72 248–254. 10.1006/abio.1976.9999 942051

[B6] Cheng F. Chen H. Lei N. Zhang M. Wan H. Shu G. (2019). Effect of prebiotics, inorganic salts and amino acids for cell envelope proteinase production from *Lactobacillus plantarum* LP69. *Acta Sci. Polon. Technol. Aliment.* 18 269–278. 10.17306/J.AFS.0656 31569909

[B7] Converti A. Perego P. Del Borghi M. (2003). Effect of specific oxygen uptake rate on *Enterobacter aerogenes* energetics: carbon and reduction degree balances in batch cultivations. *Biotechnol. Bioeng.* 82 370–377. 10.1002/bit.10570 12599264

[B8] Egnatchik R. A. Leamy A. K. Jacobson D. A. Shiota M. Young J. D. (2014). ER calcium release promotes mitochondrial dysfunction and hepatic cell lipotoxicity in response to palmitate overload. *Mol. Metab.* 3 544–553. 10.1016/j.molmet.2014.05.004 25061559 PMC4099508

[B9] He L. Xiao Y. Gebreselassie N. Zhang F. Antoniewiez M. R. Tang Y. J. (2014). Central metabolic responses to the overproduction of fatty acids in *Escherichia coli* based on ^13^C-metabolic flux analysis. *Biotechnol. Bioeng.* 111 575–585. 10.1002/bit.25124 24122357 PMC5901677

[B10] Hon M. K. Mohamad M. S. Mohamed Salleh A. H. Choon Y. W. Mohd Daud K. Remli M. A. (2019). Identifying a gene knockout strategy using a hybrid of simple constrained artificial bee colony algorithm and flux balance analysis to enhance the production of succinate and lactate in *Escherichia coli*. *Interdiscipl. Sci. Comput. Life Sci.* 11 33–44. 10.1007/s12539-019-00324-z 30758766

[B11] Hong S. H. Lee S. Y. (2001). Metabolic flux analysis for succinic acid production by recombinant *Escherichia coli* with amplified malic enzyme activity. *Biotechnol. Bioeng.* 74 89–95. 10.1002/bit.1098 11369997

[B12] Huang B. Qin P. Xu Z. Zhu R. Meng Y. (2011). Effects of CaCl_2_ on viscosity of culture broth, and on activities of enzymes around the 2-oxoglutarate branch, in *Bacillus subtilis* CGMCC 2108 producing poly-(γ-glutamic acid). *Bioresour. Technol.* 102 3595–3598. 10.1016/j.biortech.2010.10.073 21071211

[B13] Huang J. F. Liu Z. Q. Jin L. Q. Tang X. L. Shen Z. Y. Yin H. H. (2017). Metabolic engineering of *Escherichia coli* for microbial production of L-methionine. *Biotechnol. Bioeng.* 114 843–851. 10.1002/bit.26198 27723097

[B14] Huang J. F. Shen Z. Y. Mao Q. L. Zhang X. M. Zhang B. Wu J. S. (2018). Systematic analysis of bottlenecks in a multibranched and multilevel regulated pathway: the molecular fundamentals of L-methionine biosynthesis in *Escherichia coli*. *ACS Synth. Biol.* 7 2577–2589. 10.1021/acssynbio.8b00249 30274509

[B15] Jacob J. Rajendran R. U. Priya S. H. Purushothaman J. Saraswathy Amma D. K. B. N. (2017). Enhanced antibacterial metabolite production through the application of statistical methodologies by a *Streptomyces nogalater* NIIST A30 isolated from western ghats forest soil. *PLoS One* 12:e0175919 10.1371/journal.pone.0175919 PMC5402949 28437452

[B16] Kaleta C. Schäuble S. Rinas U. Schuster S. (2013). Metabolic costs of amino acid and protein production in *Escherichia coli*. *Biotechnol. J.* 8 1105–1114. 10.1002/biot.201200267 23744758

[B17] Kase H. Nakayama K. (1975). L-Methionine production by methionine analog-resistant mutants of *Corynebacterium glutamicum*. *Agric. Biol. Chem.* 39 153–160. 10.1271/bbb1961.39.153

[B18] Kind S. Becker J. Wittmann C. (2013). Increased lysine production by flux coupling of the tricarboxylic acid cycle and the lysine biosynthetic pathway - Metabolic engineering of the availability of succinyl-CoA in *Corynebacterium glutamicum*. *Metab. Eng.* 15 184–195. 10.1016/j.ymben.2012.07.005 22871505

[B19] Klamt S. Muller S. Regensburger G. Zanghellini J. (2018). A mathematical framework for yield (vs. rate) optimization in constraint-based modeling and applications in metabolic engineering. *Metab. Eng.* 47 153–169. 10.1016/j.ymben.2018.02.001 29427605 PMC5992331

[B20] Koffas M. Roberge C. Lee K. Stephanopoulos G. (1999). Metabolic engineering. *Ann. Rev. Biomed. Eng.* 1 535–557. 10.1146/annurev.bioeng.1.1.535 11701499

[B21] Krömer J. O. Wittmann C. Schröder H. Heinzle E. (2006). Metabolic pathway analysis for rational design of L-methionine production by *Escherichia coli* and *Corynebacterium glutamicum*. *Metab. Eng.* 8 353–369. 10.1016/j.ymben.2006.02.001 16621639

[B22] Kumar D. Garg S. Bisaria V. S. Sreekrishnan T. R. Gomes J. (2003). Production of methionine by a multi-analogue resistant mutant of *Corynebacterium lilium*. *Process Biochem.* 38 1165–1171. 10.1016/S0032-9592(02)00287-X

[B23] Li X. Deng Y. Yang Y. Wei Z. Cheng J. Cao L. (2017). Fermentation process and metabolic flux of ethanol production from the detoxified hydrolyzate of cassava residue. *Front. Microbiol.* 8:1603 10.3389/fmicb.2017.01603 PMC5572243 28878755

[B24] Liu L. Li Y. Zhu Y. Du G. Chen J. (2007). Redistribution of carbon flux in *Torulopsis glabrata* by altering vitamin and calcium level. *Metab. Eng.* 9 21–29. 10.1016/j.ymben.2006.07.007 17008113

[B25] Livak K. J. Schmittgen T. D. (2001). Analysis of relative gene expression data using real-time quantitative PCR and the 2(-Delta Delta C(T)) Method. *Methods* 25 402–408. 10.1006/meth.2001.1262 11846609

[B26] Martinez-Monge I. Albiol J. Lecina M. Liste-Calleja L. Miret J. Sola C. (2019). Metabolic flux balance analysis during lactate and glucose concomitant consumption in HEK293 cell cultures. *Biotechnol. Bioeng.* 116 388–404. 10.1002/bit.26858 30411322

[B27] Morales Y. Bosque G. Vehí J. Picó J. Llaneras F. (2016). PFA toolbox: a MATLAB tool for metabolic flux analysis. *BMC Syst. Biol.* 10:46 10.1186/s12918-016-0284-1 PMC4940746 27401090

[B28] Noftsger S. St-Pierre N. R. Sylvester J. T. (2005). Determination of rumen degradability and ruminal effects of three sources of methionine in lactating cows. *J. Dairy Sci.* 88 223–237. 10.3168/jds.S0022-0302(05)72680-1 15591385

[B29] Oh Y. K. Park S. Seol E. H. Kim S. H. Kim M. S. Hwang J. W. (2008). Carbon and energy balances of glucose fermentation with hydrogenproducing bacterium *Citrobacter amalonaticus* Y19. *J. Microbiol. Biotechnol.* 18 532–538. 18388473

[B30] Oraei M. Razavi S. H. Khodaiyan F. (2018). Optimization of effective minerals on riboflavin production by *Bacillus subtilis subsp. subtilis ATCC* 6051 using statistical designs. *Avicen. J. Med. Biotechnol.* 10 49–55. PMC5742654 29296267

[B31] Papp B. Simeonidis E. (2007). Flux balance analysis and its applications. *BMC Syst. Biol.* 1:77 10.1186/1752-0509-1-S1-P77

[B32] Park S. D. Lee J. Y. Sim S. Y. Kim Y. Lee H. S. (2007). Characteristics of methionine production by an engineered *Corynebacterium glutamicum* strain. *Metab. Eng.* 9 327–336. 10.1016/j.ymben.2007.05.001 17604670

[B33] Pera L. M. Callieri D. A. (1997). Influence of calcium on fungal growth, hyphal morphology and citric acid production in *Aspergillus niger*. *Folia Microbiol.* 42 551–556. 10.1007/bf02815463 9438355

[B34] Pera L. M. Callieri D. A. (1999). Influence of calcium on fungal growth and citric acid production during fermentation of a sugarcane molasses-based medium by a strain of *Aspergillus niger*. *World J. Microbiol. Biotechnol.* 15 647–649. 10.1023/A:1008902724942

[B35] Quandt E. M. Gollihar J. Blount Z. D. Ellington A. D. Georgiou G. Barrick J. E. (2015). Fine-tuning citrate synthase flux potentiates and refines metabolic innovation in the Lenski evolution experiment. *eLife* 4 e09696 10.7554/eLife.09696 PMC4718724 26465114

[B36] Rawls K. D. Dougherty B. V. Blais E. M. Stancliffe E. Kolling G. L. Vinnakota K. (2019). A simplified metabolic network reconstruction to promote understanding and development of flux balance analysis tools. *Comput. Biol. Med.* 105 64–71. 10.1016/j.compbiomed.2018.12.010 30584952

[B37] Robson G. D. Wiebe M. G. Trinci A. P. J. (1991). Involvement of Ca2+ in the regulation of hyphal extension and branching in *Fusarium graminearum* A 3/5. *Exp. Mycol.* 15 263–272. 10.1016/0147-5975(91)90028-C

[B38] Santos V. E. Galdeano C. Gomez E. Alcon A. Garcia-Ochoa F. (2006). Oxygen uptake rate measurements both by the dynamic method and during the process growth of *Rhodococcus erythropolis* IGTS8: modelling and difference in results. *Biochem. Eng. J.* 32 198–204. 10.1016/j.bej.2006.09.025

[B39] Sharp J. (2013). Improved production of L-threonine in *Escherichia coli* by use of a DNA scaffold system. *Appl. Environ. Microbiol.* 79 774–782. 10.1128/AEM.02578-12 23160128 PMC3568567

[B40] Singh R. Mailloux R. J. Puiseuxdao S. Appanna V. D. (2007). Oxidative stress evokes a metabolic adaptation that favors increased NADPH synthesis and decreased NADH production in *Pseudomonas fluorescens*. *J. Bacteriol.* 189 6665–6675. 10.1128/JB.00555-07 17573472 PMC2045160

[B41] Srere P. A. (1969). [1] Citrate synthase: [EC 4.1.3.7. Citrate oxaloacetate-lyase (CoA-acetylating). *Methods Enzymol.* 13 3–11.

[B42] Stephanopoulos G. Sinskey A. J. (1993). Metabolic engineering — methodologies and future prospects. *Trends Biotechnol.* 11 392–396. 10.1016/0167-7799(93)90099-U 7764086

[B43] Themelis D. G. Tzanavaras P. D. Anthemidis A. N. Stratis J. A. (1999). Direct, selective flow injection spectrophotometric determination of calcium in wines using methylthymol blue and an on-line cascade dilution system. *Analyt. Chim. Acta* 402 259–266. 10.1016/S0003-2670(99)00503-6

[B44] Tran L. M. Rizk M. L. Liao J. C. (2008). Ensemble modeling of metabolic networks. *Biophys. J.* 95 5606–5617. 10.1529/biophysj.108.135442 18820235 PMC2599852

[B45] Usuda Y. Kurahashi O. (2005). Effects of deregulation of methionine biosynthesis on methionine excretion in *Escherichia coli*. *Appl. Environ. Microbiol.* 71 3228–3234. 10.1128/AEM.71.6.3228-3234.2005 15933025 PMC1151843

[B46] Vallino J. J. Stephanopoulos G. (1993). Metabolic flux distributions in *Corynebacterium glutamicum* during growth and lysine overproduction. *Biotechnol. Bioeng.* 41 633–646. 10.1002/bit.260410606 18609599

[B47] Varma A. Palsson B. O. (1994). Metabolic flux balancing: basic concepts, scientific and practical use. *Nat. Biotechnol.* 12 994–998. 10.1038/nbt1094-994

[B48] Wang Y. San K.-Y. Bennett G. N. (2013). Cofactor engineering for advancing chemical biotechnology. *Curr. Opin. Biotechnol.* 24 994–999. 10.1016/j.copbio.2013.03.022 23611567

[B49] Wiesenberger G. Steinleitner K. Malli R. Graier W. F. Vormann J. Schweyen R. J. (2007). Mg^2 +^ deprivation elicits rapid Ca^2 +^ uptake and activates Ca^2 +^/calcineurin signaling in *Saccharomyces cerevisiae*. *Eukaryot. Cell* 6 592–599. 10.1128/ec.00382-06 17337637 PMC1865649

[B50] Xin X. Wang Y. (2007). Ultrastructural and intracellular chemical changes of a novel alophilic strain V430 of *Staphylococcus saprophyticus* under CaCl_2_ stress. *Appl. Biochem. Biotechnol.* 142 298–306. 10.1007/s12010-007-0038-z 18025590

[B51] Xu J. Z. Yang H. K. Zhang W. G. (2018). NADPH metabolism: a survey of its theoretical characteristics and manipulation strategies in amino acid biosynthesis. *Crit. Rev. Biotechnol.* 38 1061–1076. 10.1080/07388551.2018.1437387 29480038

[B52] Yin L. Zhao J. Cheng C. Hu X. Wang X. (2014). Enhancing the carbon flux and NADPH supply to increase L-isoleucine production in *Corynebacterium glutamicum*. *Biotechnol. Bioprocess Eng.* 19 132–142. 10.1007/s12257-013-0416-z

[B53] Zhao X. Kasbi M. Chen J. Peres S. Jolicoeur M. (2017). A dynamic metabolic flux analysis of ABE (acetone-butanol-ethanol) fermentation by *Clostridium acetobutylicum* ATCC 824, with riboflavin as a by-product. *Biotechnol. Bioeng.* 114 2907–2919. 10.1002/bit.26393 28853155

[B54] Zhou H. Y. Wu W. J. Niu K. Xu Y. Y. Liu Z. Q. Zheng Y. G. (2019). Enhanced L-methionine production by genetically engineered *Escherichia coli* through fermentation optimization. *3 Biotech.* 9:96 10.1007/s13205-019-1609-8 PMC6385071 30800607

[B55] Zhou W. Liu X. Zhang P. Zhou P. Shi X. (2014). Effect analysis of mineral salt concentrations on nosiheptide production by *Streptomyces actuosus* Z-10 using response surface methodology. *Molecules* 19 15507–15520. 10.3390/molecules191015507 25264834 PMC6270855

