# Supplementary material for: Calcium Carbonate Addition Improves L-Methionine Biosynthesis by Metabolically Engineered Escherichia coli W3110-BL
Source: Front Bioeng Biotechnol. 2020 Apr 24;8:300. doi: 10.3389/fbioe.2020.00300 (PMC7212366; doi:10.3389/fbioe.2020.00300)
Supplement: Supplementary file 1 [file Table_1.DOCX]

**Supplementary Table 1.** The primers for qRT-PCR of the genes associated with TCA cycle

| **Enzyme** | **Gene** | **Primer sequence (5**′**-3**′**)** |
| --- | --- | --- |
| - | 16sRNA | rRNA-F: GCCACACTGGAACTGAGACA |
|  |  | rRNA-R: TCCTCCCCGCTGAAAGTA |
| Citrate synthase | *gltA* | gltA-F: TACTCTCGGTTCAAAAGGTGTGTTC |
|  |  | gltA-R: GCAGCAAAATACCTTCATCACCATC |
| Aconitase | *acnA* | acnA-F: AAGGTCGGGGTGTTGAGCGAAAAGA |
|  |  | acnA-R: CACCACACGAATACCAAGCAGACGC |
|  | *acnB* | acnB-F: CAACGAATATGCGAAGCAGGTTATG |
|  |  | acnB-R: GGTCTGGCTCAATACCTTCACGGGC |
| Isocitrate dehydrogenase | *icdA* | icdA-F: GTGATGGAATCGGTGTAGATGTAAC |
|  |  | icdA-R: ATTTCCATCCAGGAGATTTTACGCT |
| α-ketoglutarate dehydrogenase | *sucA* | sucA-F: TGGTTGGACTCTTCTTACCTCTCTG |
|  |  | sucA-R: TTCACATTGGTGTCAGGGTCGGAGA |
|  | *sucB* | sucB-F: CCACCGTCGCAACCTGGCATAAAAA |
|  |  | sucB-R: TGGACGCTTTCTCTTCAGATTTGGC |
| Succinyl-CoA synthetase | *sucC* | sucC-F: AACTTTTTGCCCGCTATGGCTTACC |
|  |  | sucC-R: CCTGACATTTCACTACCCACGGACC |
|  | *sucD* | sucD-F: AGGGGACTTTCCACTCAGAACAGGC |
|  |  | sucD-R: GGATGCCTTCAGTGATGGTGATAAT |
| Succinate dehydrogenase | *sdhA* | sdhA-F: CTGTGAATGAGAAAGGCGAAGATGT |
|  |  | sdhA-R: TCACGGAAGACCGAGAAGTTATGCT |
|  | *sdhB* | sdhB-F: GGATTACACCCTGGAAGCGGATGAA |
|  |  | sdhB-R: TTCACGGCAGGAGCGGCGGAACGAC |
|  | *sdhC* | sdhC-F: TCCCCATCACGGCGATAGCGTCCAT |
|  |  | sdhC-R: CGTGATACGCCAGAGCGGTAAGGAT |
|  | *sdhD* | sdhD-F: AGCAACGCCTCCGCATTAGGACGCA |
|  |  | sdhD-R: TGAACGCAGAGGCGAAGAAACCGAT |
| Fumarase | *fumA* | fumA-F: TTTATGCCGTTGATGGCGACGAATA |
|  |  | fumA-R: GAACGCAATATGATACGGAGGACAG |
|  | *fumB* | fumB-F: AACCTGAAAACCGTCAAGTTAGCAA |
|  |  | fumB-R: GGTCAACTTTCACCGCTTCGCCTTC |
| Malate dehydrogenase | *Mdh* | Mdh-F: TTTTTCTGGTGAAGATGCGACTCCG |
|  |  | Mdh-R: CTGGCTGTTTGCCTTTCAGTTCCGC |

All the primers were synthesized by Zhejiang Shangya Biotechnology Co., Ltd. (Hangzhou, China)

**Supplementary Table 2.** The abbreviations of metabolites

| Abbreviation | Full name |
| --- | --- |
| GLC | Glucose |
| ATP | Adenosine triphosphate |
| NADH | Nicotinamide adenine dinucleotide |
| NADPH | Nicotinamide adenine dinucleotide phosphate |
| FADH_2_ | Flavine adenine dinucleotide, reduced |
| G6P | Glucose 6-phosphate |
| F6P | Fructose-6-phosphate |
| 3PG | Glyceraldehyde-3-phosphate |
| PEP | Phosphoenolpyruvate |
| PYR | Pyruvate |
| CoA | Coenzyme A |
| AcCoA | Acetyl-CoA |
| CIT | Citrate |
| RL5P | Ribulose 5-phosphate |
| X5P | Xylulose-5-phosphate |
| R5P | Ribose-5-phosphate |
| S7P | Sedoheptulose-7-phosphate |
| GAP | Glyceraldehyde-3-phosphate |
| E4P | Erythrose-4-phosphate |
| FOR | Formate |
| α-KG | α-Ketoglutaric acid |
| SUC | Succinate |
| FUM | Fumarate |
| OXA | Oxaloacetate |
| Asp | Aspartate |
| Hom | Homoserine |
| Thr | Threonine |
| Glu | Glutamate |
| Gln | Glutamine |
| Ala | Alanine |
| Cys | Cysteine |
| Gly | Glycine |
| Leu | Leucine |
| Met | Methionine |
| Phe | Phenylalanine |
| Tyr | Tyrosine |
| Val | Valine |
| Ser | Serine |
| Ile | Isoleucine |

**Supplementary Fig. 1.** The agitation rate and dissolved oxygen in batch runs with different pH control modes

**Symbols:**

Agitation rate: **——** Control; **——** 10 g/L CaCO_3_; **——** 20 g/L CaCO_3_; **——** 30 g/L CaCO_3_; **——** 40 g/L CaCO_3_; **——** NH_3_^.^H_2_O + 30 g/L CaCO_3_; **——** NH_3_^.^H_2_O + 33.3 g/L CaCl_2_

DO: ⸳⸳⸳⸳⸳⸳⸳⸳ Control; ⸳⸳⸳⸳⸳⸳⸳⸳ 10 g/L CaCO_3_; ⸳⸳⸳⸳⸳⸳⸳⸳ 20 g/L CaCO_3_; ⸳⸳⸳⸳⸳⸳⸳⸳ 30 g/L CaCO_3_; ⸳⸳⸳⸳⸳⸳⸳⸳ 40 g/L CaCO_3_; ⸳⸳⸳⸳⸳⸳⸳⸳ NH_3_^.^H_2_O + 30 g/L CaCO_3_; ⸳⸳⸳⸳⸳⸳⸳⸳ NH_3_^.^H_2_O + 33.3 g/L CaCl_2_
